# Vascular Surgery Trainees Still Need to Learn How to Sew: Importance of Learning Surgical Techniques in the Era of Endovascular Surgery

**DOI:** 10.3389/fsurg.2015.00016

**Published:** 2015-05-11

**Authors:** Faisal Aziz

**Affiliations:** ^1^Pennsylvania State University College of Medicine, Hershey, PA, USA

**Keywords:** vascular surgery training, open surgery training, endovascular procedures, vascular surgery, endovascular

## Abstract

Vascular surgery represents one of the most rapidly evolving specialties in the field of surgery. It was merely 100 years ago when Dr. Alexis Carrel described vascular anastomosis. Over the course of next several decades, vascular surgeons distinguished themselves from general surgeons by horning the techniques of vascular surgery operations. In the era of minimally invasive interventions, the number of endovascular interventions performed by vascular surgeons has increased exponentially. Vascular surgery trainees in the current times spend considerable time in mastering the techniques of endovascular operations. Unfortunately, the reduction in number of open surgical operations has lead to concerns in regards to adequacy of learning open surgical techniques. In future, majority of vascular interventions will be done with minimally invasive techniques. Combination of poor training in open operations and increasing complexity of open surgical operations may lead to poor surgical outcomes. It is the need of the hour for vascular surgery trainees to realize the importance of learning and mastering open surgical techniques. One of the most distinguishing features of contemporary vascular surgeons is their ability to perform both endovascular and open vascular surgery operations, and we should strive to maintain our excellence in both of these arenas.

Vascular surgery is a rapidly evolving field. It was barely 100 years ago when Alexis Carrel described the technique of vascular anastomosis, which gave birth to the field of vascular surgery. Over the past few decades, vascular surgeons have horned the technical details of open vascular surgery operations. We are now entering an era, where a new generation of endovascular-trained vascular surgery is emerging. These surgeons will be highly trained individuals in the field of endovascular surgery. However, they will have less experience in performing open operations. It is a critical time for vascular surgery trainees to realize the importance of having open surgical skills in the era of ever increasing endovascular operations.

Since the introduction of endovascular technology, past two decades have seen a revolution in the field of endovascular surgery. Patients’ increasing preference for minimally invasive operations and relative ease of learning endovascular techniques have made them attractive for both patients and health care providers equally. For example, the endovascular interventions for lower extremity peripheral arterial disease have increased by more threefold over a 10-year time period, while the number of open surgical bypasses for PAD decreased by 42% (1). In fact, the rate of increase in the peripheral endovascular interventions has actually surpassed the decrease in the number of open surgical bypasses, contributing to overall doubling of the lower extremity revascularization procedures over the decade. Needless to say, this change in paradigm has also affected vascular surgery training. In USA, all medical and surgical training programs go through an accreditation process by Accreditation for Graduate Medical Education (ACGME).

A recent review of ACGME data (2) showed that over a span of 6 years, the number of endovascular procedures performed by surgical trainees increased by 422%. The number of open aortic surgeries reduced by 17% and the number of endovascular aortic aneurysm repairs (EVAR) increased by 299%. Initially, it was perceived that introduction of endovascular surgery lead surgeons to treat simpler aneurysms with endovascular technique, hence leaving more complex anatomy aneurysms to be treated with open repair and therefore an opportunity for surgical trainees to learn complex open surgical skills (3). However, with the passage of time, even more and more complex aneurysms are now being treated with endovascular techniques and hence further enhancing endovascular techniques, and indirectly causing a decline in learning open surgical technique. With increasing number of endovascular interventions, the art of open vascular surgery is seriously facing a big threat – a threat, which it has never encountered before. Statistics clearly show that vascular surgery trainees of current era will be performing fewer and fewer open vascular surgery operations in the future. This fact has serious implications: the patients who will need open surgical operations will be fewer in number, and will have higher complexity disease than majority of the patients. Unfortunately, the surgeons who will be treating these patients with difficult anatomy and complex physiology will not have enough experience of open operations. The combination of high-risk surgical patients and less experienced surgeons will likely increase the risk of perioperative complications and has a potential to impact the outcomes in a negative fashion.

Realizing the impact of decreased operative exposure and reduced number of duty hours; many centers are now actively exploring the role of simulation techniques for learning open vascular surgery techniques. A recent study by Sigounas et al. (4) evaluating the simulation for vascular anastomosis technique for surgical trainees showed a significant improvement in the surgical technique after attending the simulation course. Likewise, there are other studies, which have shown an improvement in surgical technique after exposure to simulation. It is quite possible that the future vascular surgery training programs would like to utilize simulation routinely during surgical training.

Like training in any other surgical specialty, training in vascular surgery involves repeated exposure to same operations, over and over again, so that the trainee can learn the skills by observing and assisting the senior surgeon. Learning how to operate is a slow and steady process: it takes years to gradually learn operating on complex structures inside human body. It takes even longer to learn the pearls of wisdom: when to operate, what operation to offer, and more importantly, when not to operate and how to steer one’s way out of trouble. Due to the nature of this specialty, vascular surgery patients in general are very frail and do not have much reserve to tolerate the trauma of the operation to human body. Vascular surgeons have to learn how to be careful during the conduct of the operation, and to have back-up options in mind. It is not uncommon that surgeons change their operative plans during the operation, due to patients’ anatomy and response to anesthetic and variable hemodynamics. Sometimes, the most technically feasible operation may not be the best option due to a specific patient’s condition. The complex decision making process under stressful situations demands wisdom, and unfortunately, experience is the most logical way to achieve excellence in these highly stressful situations.

## Conflict of Interest Statement

The author declares that the research was conducted in the absence of any commercial or financial relationships that could be construed as a potential conflict of interest.
